# Homozygous sickle cell disease related mortality in Senegal (2011–2020)

**DOI:** 10.1002/jha2.273

**Published:** 2021-10-04

**Authors:** Moussa Seck, Oumou Ba, Blaise Felix Faye, Sokhna Aissatou Touré, Youssou Bamar Guèye, Nata Dieng, Abibatou Sall, Macoura Gadji, Awa Oumar Touré, Saliou Diop

**Affiliations:** ^1^ Hematology Department Cheikh Anta Diop University Dakar Senegal; ^2^ National Blood Transfusion Center Clinical Hematology Dakar Senegal

**Keywords:** homozygous sickle cell disease, acute anemia, acute chest syndrome, Senegal

## Abstract

Homozygous sickle cell disease (HSCD) is characterized by multiorgan morbidity and an increased risk of early death. We aim to describe the mortality rate, causes, and risk factors of death in HSCD between 2011 and 2020. We conducted a retrospective study with a duration of 10 years in the cohort of 2348 HSCD patients. The mortality rate was determined by reporting the number of deaths to the total number of patients followed in the year. Sociodemographic, clinical, biological data and causes of death were studied. Death risk factors were determined by a bivariate analysis comparing deceased and living HSCD patients. The mean age of death was 26 years (3–52). The sex ratio was 1.2. The mortality rate was 2.76%. The death rate was high in 2011 (3.2%) and low in 2020 (0.17%). We observed a significant reduction of mortality of 94.6%. Most of the common causes of death were acute anemia (40%), acute chest syndrome (24.6%), and infections (20%). Risk factors of death were age, vaso‐occlusive crises ≥3, acute chest syndrome, blood transfusion, and chronic complications. Mortality among HSCD has significantly decreased over the past 10 years in Senegal, and the main causes of death were acute anemia, acute chest syndrome, and infections.

## 1 INTRODUCTION

Homozygous sickle cell disease (HSCD) is characterized by very high morbidity and mortality especially in Africa due to diagnosis delay and precarious means of care [1]. This morbidity is expressed by the occurrence of acute complications witch the most formidable and often causes of death are acute anemia, acute chest syndrome, infections, stroke, and chronic degenerative complications of fatal such as chronic renal failure, heart failure, and pulmonary artery hypertension [2, 3].

In recent years, improving the management of HSCD allowed an extension of life expectancy as well as in developed countries [4] than in developing ones [5, 6]. This dramatic improvement has been attributed to several management during early childhood, including neonatal screening, antibiotic prophylaxis [7], pneumococcal vaccination [8], and hydroxyurea which has been shown to be effective in HSCD treatment reducing morbidity and mortality [9, 10, 11].

Several studies have evaluated mortality associated with sickle cell disease in recent years, and in most of these studies risk factors associated with death were identified [12 –14]. In Senegal, the only study that evaluated mortality in HSCD dates back to 2003 and involved a small cohort of 108 HSCD patients followed on average for 5 years, showed a death rate of 4.6% [5]. In this view, it is necessary to study mortality, causes, and risk factors of death on a larger cohort of HSCD patients.

## 2 METHODS

We realized a retrospective study including 65 HSCD patients who died of complications related to HSCD between 2011 and 2020 of a cohort of 2348 HSCD patients diagnosed by alkaline pH hemoglobin electrophoresis and regularly followed with at least two consultations per year. Each patient had a medical folder in which sociodemographic, clinical, biological, and therapeutic data were recorded. The mortality rate per year was determined by reporting the number of deaths out of the total number of patients followed in the same year. Determining the death rate per year has made it possible to follow mortality evolution over the past 10 years. The causes of death were clinically evaluated and corresponded to the clinical event immediately preceding patient's death.

Other parameters were sociodemographic (age, sex, duration of follow‐up) and HSCD morbidity data including age at diagnosis, number of vaso‐occlusive crises (VOC) per year, acute complications such as infections, acute anemia, acute chest syndrome, stroke and chronic complications consisting of heart failure, chronic renal failure, and pulmonary arterial hypertension. The risk factors for death were studied by a bivariate analysis comparing deceased and living HSCD patients with a significant *p*‐value less than 0.05. The data were analyzed by EPI info software version 3.5.4.

## 3 RESULTS

### 3.1 Characteristics baseline of HSCD death patients

The sex ratio (M/F) was 1.2. The mean age was 26 years (3–52): 25.7 years (11–52) for men and 26.5 years (3–50) for women. The duration of follow‐up was 16 years (1–31). The history of hospitalizations was 53.8%, and transfusion history was 78.4%. According to acute complications, the mean number of VOC/year was 2 (1–5), acute anemia (47.7%), acute chest syndrome (6.1%), stroke (3%), serious infections (29.2%) consisting of bacterial septicemia, malaria and tuberculosis, and priapism (16.9%). Chronic complications were 95.3% including femoral osteonecrosis (26%), chronic renal failure (24%), heart failure (22%), and biliary lithiasis (22%). HSCD patient's chronic transfusion program was 9.2%. No patient was treated by hydroxyurea.

### 3.2 Mortality, causes of death, mean age, and sex ratio of HSCD patients

**FIGURE 1:**
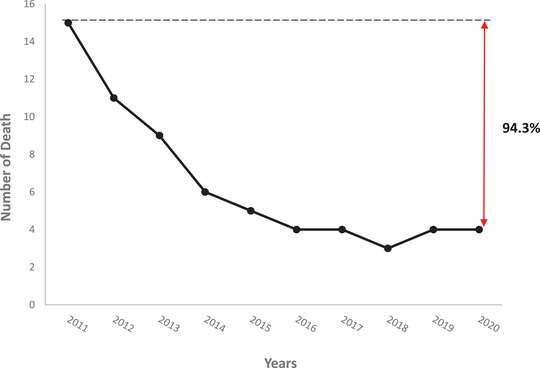
Mortality rate decrease from 2011 to 2020

Between 2011 and 2020, the global mortality was 2.76% (65 deaths for 2348 HSCD patients followed). The death rate per year was high in 2011 (3.2%) and low in 2020 (0.17%) despite an increase in the number of patients from 460 (2011) to 2348 patients (2020) (Table 1). We observed that the death rate was decreased by 94.6% over the past 10 years (Figure 1). The leading causes of death consisted of acute anemia (40%) (mean age: 25.2 years, sex ratio: 1.6), acute chest syndrome (24.6%) (mean age: 25.3 years, sex ratio: 0.6), and serious infections (20%) (mean age: 22.3 years, sex ratio: 3.3) (Table 2).

**TABLE 1:** Mortality and number of HSCD patients followed per year (2011–2020)

	Patients followed	Number of death	Rate of death (%)
Years	(*N* = 2348)	(*n* = 65)	(2.76%)
2011	460	15	3.2
2012	514	11	2.1
2013	818	09	1.1
2014	1037	06	0.6
2015	1277	05	0.4
2016	1457	04	0.27
2017	1746	04	0.22
2018	1974	03	0.15
2019	2190	04	0.18
2020	2348	04	0.17

**TABLE 2:** Causes of death, number of deaths, mean age, and sex ratio

Causes of death	Number of death (*n*, %)	Mean age (years)	Sex ratio
Acute anemia	26 (40%)	25.2	1.6
Acute chest syndrome	16 (24.6%)	25,3	0.6
Serious infections	13 (20%)	22.3	3.3
Chronic renal failure	6 (9.2%)	32.3	0.5
Ischemic strokes	2 (3.1%)	20	1
Biliary lithiasis	2 (3.1%)	29.5	1

### 3.3 Risk factors for death in HSCD patients

The factors significantly associated with death were age between 20 and 40 years, the number of VOC ≥3, acute chest syndrome, blood transfusion history, and chronic complications (Table 3).

**TABLE 3:** Risk factors for death in HSCD patients

	HSCD death	HSCD followed	
Parameters	(*n* = 65)	(*N *= 2283)	*p*
Age			
< 20 years	5 (7,.7%)	271 (11.9%)	0.15
20 – 40 years	53 (81.5%)	1399 (61.3%)	**0.0019**
> 40 years	7 (10.8%)	611 (26.8%)	0.074
Sex			
Men	36 (55.3%)	1059 (46.4%)	0.07
Femel	29 (44.6%)	1223 (53.6%)	0.08
Vaso‐occlusive crisis/years (≥3)	40 (61.5%)	999 (43.8%)	**0.0026**
Acute anemia	31 (47.7%)	1326 (58.1%)	0.097
Acute chest syndrome	4 (6.1%)	1940 (0,85%)	**<0.00001**
Ischemic strokes	2 (3%)	48 (2,12%)	0.39
Serious infections	19 (29.2%)	607 (26.6%)	0.35
History of hospitalizations	35 (53.8%)	1011 (44.3%)	0.074
History of transfusion	51 (78.4%)	650 (28.5%)	**<0.00001**
Chronic complications	62 (95.3%)	910 (39.9%)	**<0.00001**

Statistically significant values are put in bold.

## 4 DISCUSSION

We show through this study that the death rate in HSCD is 2.76% over a period of 10 years, and that acute anemia, acute chest syndrome, and infections are the main causes of death. We also note that the mortality is gradually decreased from 3.2% (2011) to 0.17% (2020). This result is lower than that reported in a previous study where the mortality rate was 4.6% [5]. This reduction in mortality of HSCD is due to the progress in improving the care of patients with the reduction of mean age at diagnosis, early care in hematology, antibiotic prophylaxis in children up to 5 years old, opening clinical hematology for the early management of HSCD emergencies and the organization of a multidisciplinary follow in Senegal.

We note a higher mortality rate in men with a sex ratio of 1.2 but without a significant difference in the mean age of death (25.7 years for men and 26.5 years for women). Although HSCD is responsible for a significant excess risk of maternal death mainly due to serious complications of HSCD [15].

Nevertheless, mortality in HSCD remains high in Africa and particularly in children under 5 years [6]. In developed countries, this mortality is low because of the progress related to newborn screening, hydroxyurea treatment, long‐term transfusion programs, and allogeneic bone marrow transplantation [7, 11, 16].

The main causes of death were acute anemia, acute chest syndrome, and infections. Three main etiologies worsening anemia in HSCD are hyperhemolysis, splenic sequestration occurring in children under 5 years, and acute erythroblastopenia due to parvovirus B19. In our series, acute anemia was mainly related to hyperhemolysis caused by an infectious disease such as malaria, sepsis, and tuberculosis and prolonged VOC. In Nigeria, a study shows a lower rate of death from acute anemia (31%), of which splenic sequestration was the main cause of death [17]. However, in the United States, only 1.36% of HSCD deaths were due to splenic sequestration [18]. This high mortality linked to acute anemia in Africa is due to the management delay which can be explained by a diagnosis delay and the unavailability of red blood cell concentrates in extreme emergency [1].

Acute chest syndrome was the second cause of death in our study, compared to developed countries, where it is the leading cause of HSCD death, but at lower rates around 5% of deaths [19]. This difference can be explained by the diagnosis difficulties of acute chest syndrome and the delay in treatment in Africa. Acute chest syndrome related mortality is due to the rapid onset symptoms of chest tightness causing premature HSCD death if emergency care is not begun. Oxygen therapy and single or exchange blood transfusion are the mainstays of treatment of acute chest syndrome [20, 21]. Several studies demonstrated the benefit of transfusion exchange with a 40% reduction in mortality because it improves microvascular perfusion and increases oxygen transport capacity [22, 23].

Infectious complications represent one of the most frequent complications in HSCD with a peak frequency in children [24]. They constituted 78% of the causes of death among SCD in Africa [25] and 45% of deaths in Brazil [26]. In our study, infections are responsible for 20% of HSCD deaths. They were mainly due to malaria and pulmonary infections, while in Nigeria meningitis is predominantly responsible for 13.2% of HSCD deaths [27].

Other causes of HSCD deaths consisted of chronic renal failure, ischemic stroke, and cholecystectomy complications. Sickle cell nephropathy appears increasingly as a common complication and has long‐term serious consequences for patient survival [14, 28]. Chronic renal failure in HSCD was responsible for 9.2% of deaths, lower than in developed countries [22]. It has a poor prognosis in Africa due to the unavailability of renal transplantation and little easy access to symptomatic treatment by dialysis [29]. Microalbuminuria assay should be performed regularly in HSCD anemia to detect renal failure to start early treatment before kidney failure sets in.

Strokes are common in HSCD especially in childhood and are responsible for high mortality reaching over 25% [26].

HSCD deaths associated with biliary lithiasis were associated with acute cholecystitis, but especially postsurgical complications of cholecystectomy. The risk of death is real in the immediate aftermath of a cholecystectomy, hence the importance of strict and adequate monitoring [30].

Other HSCD chronic complications related to cardiovascular injuries such as heart failure and pulmonary hypertension are often responsible for HSCD deaths [31]. Other studies have shown the influence of severe anemia with an increase in HSCD deaths [32].

The risk factors associated with death were age between 20 and 40 years, the number of VOC ≥3, acute chest syndrome, priapism, blood transfusion history, and chronic complications. Most of these factors have already been reported in other studies except priapism [12, 13].

## 5 CONCLUSION

Mortality among HSCD has significantly decreased over the past 10 years in Senegal reflecting the progress in HSCD management, and the main causes of death were acute anemia, acute chest syndrome, and infections. We, therefore, recommend early diagnosis and emergency management of these acute complications, particularly, in Africa.

## References

[1] Diop S , Pirenne F . Transfusion and sickle cell anemia in Africa. Transf Clin Biol. 2012;28(2):143‐5 10.1016/j.tracli.2021.01.013 33515732

[2] McClellan Ann C , Jean‐Christophe L , Debaun RM . High one year mortality in adults with sickle cell disease and end‐stage renal disease. Br J Haematol. 2012;159(3):360‐7 22967259 10.1111/bjh.12024 PMC4562224

[3] Shruti C , Djamila LG , Natalie J , Adetola K , Mark R , Michael RD . Clustering of end‐organ disease and earlier mortality in adults with sickle cell disease: A retrospective‐prospective cohort study. Am J Hematol. 2018;93:1153–60. 29981283 10.1002/ajh.25202

[4] Gregory JK , Frédéric BP , Clarice DR , Marilyn HG , Kwaku OF , Lakshmanan K . Sickle cell disease. Nat Rev Disease Primer. 2018;4:18010. 10.1038/nrdp.2018.10 29542687

[5] Diop S , Mokono SO , Ndiaye M , Toure Fall AO , Thiam D , Diakhaté L . La drépanocytose homozygote après l’âge de 20 ans: suivi d'une cohorte de 108 patients au CHU de Dakar. Rev Med Int. 2003;24:711‐5. 10.1016/s0248-8663(03)00220-0 14604747

[6] Scott DG , Isaac O , Hani KA , Djesika DA , Frédéric BP , Thomas NW . Sickle cell disease in Africa : A neglected cause of early childhood mortality. Am J Prev Med 2011;41(6S4):S398–S405 22099364 10.1016/j.amepre.2011.09.013 PMC3708126

[7] Rankine MAE , Owusu OS . Prophylactic antibiotics for preventing pneumococcal infection in children with sickle cell disease. Cochrane Database Syst Rev. 2017, Issue 10, Art. No.: CD003427. 10.1002/14651858.CD003427.pub4 PMC6485662 28994899

[8] Quinn CT , Rogers ZR , McCavit TL , Buchanan GR . Improved survival of children and adolescents with sickle cell disease. Blood 2010;115:3447‐52 20194891 10.1182/blood-2009-07-233700 PMC2867259

[9] Steinberg MH , McCarthy WF , Castro O , Ballas SK , Armstrong FD , Smith W , et al. The risks and benefits of long‐term use of hydroxyurea in sickle cell anemia: a 17.5 years follow‐up. Am J Hematol. 2010;85:403‐8. 20513116 10.1002/ajh.21699 PMC2879711

[10] Voskaridou E , Christoulas D , Bilalis A , Plata E , Varvagiannis K , Stamatopoulos G , et al. The effect of prolonged administration of hydroxyurea on morbidity and mortality in adult patients with sickle cell syndromes: results of a 17‐year, single‐center trial (LaSHS). Blood 2010;115:2354‐63 19903897 10.1182/blood-2009-05-221333

[11] Nevitt SJ , Jones AP , Howard J . Hydroxyurea (hydroxycarbamide) for sickle cell disease. Cochrane Database Syst Rev. 2017, Issue 4, Art. No.: CD002202. 10.1002/14651858.CD002202.pub2 PMC6478259 28426137

[12] Gladwin MT , Barst RJ , Gibbs JS , Hildesheim M , Sachdev V , Nouraie M , et al. Risk factors for death in 632 patients with sickle cell disease in the United States and United Kingdom. PLoSOne. 2014;9(7):e99489. 10.1371/journal.pone.0099489.eCollection2014 PMC4079316 24988120

[13] Poulami M , Melissa C , Laura R , Payal CD , Susan J , Mehdi N . Risk factors for mortality in adult patients with sickle cell disease: a meta‐analysis of studies in North America and Europe. Haematologica. 2017;102(4):626‐636 28104703 10.3324/haematol.2016.153791 PMC5395103

[14] Maya V , Jifang Z , David A , Jin H , Robert EM , Shivi J , et al. The morbidity and mortality of end stage renal disease in sickle cell disease. Am J Hematol. 2019;94(5):E138–E141. 30773675 10.1002/ajh.25439 PMC6449200

[15] Lesage N , Deneux TC , Saucedo M , Habibi A , Galacteros F , Girot R , et al. Maternal mortality among women with sickle‐cell disease in France, 1996–2009. Eur Obstet Gynecol Reproduct. 2015;194:183–188 10.1016/j.ejogrb.2015.09.016 26431903

[16] Adetola AK , Deva S . Hematopoietic stem cell transplantation for sickle cell disease: The changing landscape. Hematol Oncol Stem Cell Ther. 2017;10:259–266 28641096 10.1016/j.hemonc.2017.05.008

[17] Darbari DS , Kple‐Faget P , Kwangyan J , Rana S , Castro O . Circumstances of death in adult sickle cell disease patients. Am J Haematol. 2006;81(11):858‐863. 10.1002/ajh.20685 16924640

[18] Leikin SL , Gallagher D , Kinney TR , Sloane D , Klug P , Rida W . Mortality in children and adolescents with sickle cell disease. Cooperative study of sickle cell disease. Pediatrics. 1989;84(3):500‐8. 2671914

[19] Maitre B , Habibi A , Roudot‐Thoraval F , Bachir D , Belghiti DD , Galacteros F , et al. Acute chest syndrome in adults with sickle cell disease. Rev des Maladies Respir Act. 2014(2):69‐72 10.1378/chest.117.5.1386 10807826

[20] Lionnet F , Arlet JB , Bartolucci P . Recommandations pratiques de la prise en charge de la drépanocytose de l'adulte. Rev Med Int. 2009;30:S162‐S223 10.1016/j.revmed.2009.07.001 19713011

[21] Dolatkhah R , Dastgiri S . Blood transfusions for treating acute chest syndrome in people with sickle cell disease. Cochrane Database of Syst Rev. 2020, Issue 1. Art. No.: CD007843. 10.1002/14651858.CD007843.pub4 31942751 PMC6984655

[22] Allareddy V , Roy A , Lee MK , Nalliah RP , Rampa S , Rotta AT . Outcomes of acute chest syndrome in adult patients with sickle cell disease: Predictors of mortality. PLoS One. 2014;9(4):94387. doi :10.1371/journal.pone.0094387 PMC3989222 24740290

[23] Boulat C . La transfusion du drépanocytaire. Transf clin biol. 2013;20(2):68–71. 10.1016/j.tracli.2013.02.014 23597585

[24] Diop S , Diop D , Seck M , Guèye Y , Faye A , Dièye TN , et al. Facteurs prédictifs des complications chroniques de la drépanocytose homozygote chez l'adulte à Dakar (Sénégal). Med Trop. 2010;70:471‐474 21520649

[25] Lanzkron S , Carroll CP , Haywood C Jr. Mortality rates and age at death from sickle cell disease: U.S., 1979–2005. Public Health Rep. 2013;128(2):110‐6. 23450875 10.1177/003335491312800206 PMC3560868

[26] Sabarense AP , Lima GO , Silva LM , Viana MB . Survival of children with sickle cell disease in the comprehensive newborn screening programme in Minas Gerais, Brazil. Paediatr Int Child Health. 2015;35(4):329‐32 26744158 10.1080/20469047.2015.1109235

[27] Obaro SK , Tam PY . Preventing Infections in Sickle Cell Disease: The Unfinished Business. Pediatr Blood Cancer. 2016;63(5):781‐5 26840500 10.1002/pbc.25911

[28] Al Kahtani MA , AlQahtani M , Alshebaily MM , Abd Elzaher M , Moawad A , Aljohani N . Morbidity and pregnancy outcomes associated with sickle cell anemia among Saudi women. Int J Gynaecol Obstet. 2012;119(3):224‐6 22986097 10.1016/j.ijgo.2012.07.008

[29] Cazenave M , Koehl B , Nochy D , Tharaux PL , Audard V . Spectrum of renal manifestations in sickle cell disease. Nephrol Ther. 2014;10(1):10‐6. 24113202 10.1016/j.nephro.2013.07.366

[30] Manci AE , Culberson DE , Yih‐Ming Y , Gardner MT , Randall P , Johnson H , et al. Causes of death in sickle cell disease: an autopsy study. Br J Haemato. 2003;123:359‐365 10.1046/j.1365-2141.2003.04594.x 14531921

[31] Gladwin MT . Cardiovascular complications and risk of death in sickle‐cell disease. Lancet 2016;387:2565–74 27353687 10.1016/S0140-6736(16)00647-4

[32] Kenneth IA , Victor RG , Irene A , Jennifer AC , Kimberly G , Isabel EA . Low hemoglobin increases risk for cerebrovascular disease, kidney disease, pulmonary vasculopathy, and mortality in sickle cell disease: a systematic literature review and meta‐analysis. PLoS One 2020;15(4):e0229959. 10.1371/journal.pone.0229959 32243480 PMC7122773

